# Development of a fall prediction risk using multidimensional data from the Canadian Longitudinal Study on Aging

**DOI:** 10.1093/geroni/igaf122.2624

**Published:** 2025-12-31

**Authors:** Paulo Roberto Carvalho do Nascimento, Marla Beauchamp, Jinhui Ma, Luciana Macedo, Lauren Griffith

**Affiliations:** McMaster University, Hamilton, Ontario, Canada; McMaster University, Hamilton, Ontario, Canada; McMaster University, Hamilton, Ontario, Canada; McMaster University, Hamilton, Ontario, Canada; McMaster University, Hamilton, Ontario, Canada

## Abstract

Falls rank first in injury prevention priorities in Canada. Approximately 40% of falls among community-dwelling older adults could be prevented with proper strategies. This study aimed to develop a fall risk index using multidimensional data from the Canadian Longitudinal Study on Aging. We selected 36 potential fall risk factors from systematic reviews and fall guidelines. Predictor variables were extracted from baseline data of older adults (≥ 65 years, n = 12,646), while incident injurious falls were retrieved from follow-up 1 (FUP 1). At FUP1, 8.07% of the participants had an injurious fall. A stepwise multivariable logistic regression model identified 14 predictors associated with injurious falls. Significant (p < 0.05) predictors including age, previous falls, previous injurious falls, vision impairment, pain, home dissatisfaction, comorbidities, grip strength, and use of antidepressants were used to create the final model. The dataset was split into training (70%) and test (30%) datasets for model fitting and predict the probabilities, respectively. The model’s performance was modest (AUC = 0.63), with a sensitivity of 75.50%, specificity (46.77%), positive predictive value (10.07%), and negative predictive value (96.03%). Although these results limit the model’s reliability in confirming high-risk cases, it suggests that the model is more effective at ruling out individuals at low risk of injurious falls. Therefore, while the model may have limited utility for identifying those at high risk, it could be valuable as a screening tool to exclude low-risk individuals and focus preventive strategies on those more likely to experience falls.

